# Structural and Biochemical Characterization of Cysteinylation in Broadly Neutralizing Antibodies to HIV-1

**DOI:** 10.1016/j.jmb.2021.167303

**Published:** 2021-12-03

**Authors:** Oluwarotimi Omorodion, Ian A. Wilson

**Affiliations:** 1 Department of Integrative Structural and Computational Biology, The Scripps Research Institute, La Jolla, CA 92037, USA; 2 IAVI Neutralizing Antibody Center, The Scripps Research Institute, La Jolla, CA 92037, USA; 3 Consortium for HIV/AIDS Vaccine Development (CHAVD), The Scripps Research Institute, La Jolla, CA 92037, USA; 4 Skaggs Institute for Chemical Biology, The Scripps Research Institute, La Jolla, CA 92037, USA

**Keywords:** broadly neutralizing antibodies to HIV-1, CDRH3, post-translational modification, cysteinylation, antibody crystal structures

## Abstract

Antibodies with exceptional breadth and potency have been elicited in some individuals during natural HIV-1 infection. Elicitation and affinity maturation of broadly neutralizing antibodies (bnAbs) is therefore the central goal of HIV-1 vaccine development. The functional properties of bnAbs also make them attractive as immunotherapeutic agents, which has led to their production and optimization for passive immunotherapy. This process requires *in vitro* manufacturing and monitoring of any heterogeneous expression, especially when subpopulations of antibodies are produced with varying levels of biological activity. Post-translational modification (PTM) of antibodies can contribute to heterogeneity and is the focus of this study. Specifically, we have investigated cysteinylation in a bnAb lineage (PCDN family) targeting the N332-glycan supersite on the surface envelope glycoprotein (Env) of HIV-1. This PTM is defined by capping of unpaired cysteine residues with molecular cysteine. Through chromatography and mass spectrometry, we were able to characterize subpopulations of cysteinylated and non-cysteinylated antibodies when expressed in mammalian cells. The crystal structures of two PCDN antibodies represent the first structures of a cysteinylated antibody and reveal that the cysteinylation in this case is located in CDRH3. Biophysical studies indicate that cysteinylation of these HIV-1 antibodies does not interfere with antigen binding, which has been reported to occur in other cysteinylated antibodies. As such, these studies highlight the need for further investigation of cysteinylation in anti-HIV and other bnAbs.

## Highlights

• Cysteinylation is a little-studied post-translational modification in antibodies.
• Cysteinylation was identified in a lineage of bnAbs to HIV-1.
• The cysteinylation modification is clearly visualized in the crystal structures.
• Cysteinylation does not impair binding to antigen, compared to previous studies.
• These findings provide further perspectives on the role of antibody cysteinylation.

## Graphical abstract

**Figure:**
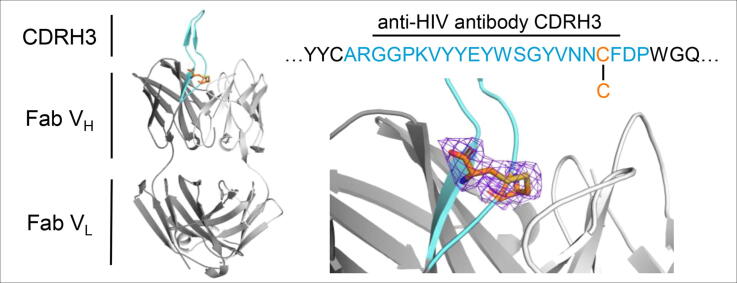

## Introduction

The discovery of broadly neutralizing antibodies (bnAbs)^1^ to the human immunodeficiency virus type 1 (HIV-1) in individuals during natural infection has revolutionized the search for an effective vaccine.^1^, ^2^, ^3^ These bnAbs function by targeting highly conserved sites on the envelope glycoprotein (Env, gp160), which is the only HIV-1 antigen on the virus surface recognized by the humoral arm of the immune system. This acquired property is especially crucial, as it allows the immune system to neutralize Env more broadly despite its high mutation rate and enormous variability in infected individuals. The isolation of bnAbs in some 10 – 15% of HIV-infected persons indicates that administration of carefully designed and selected immunogens could trigger a germline response to the virus that can be shepherded towards immune breadth via affinity maturation.^2^, ^4^, ^5^

However, these bnAbs can also have important therapeutic applications besides serving as benchmarks for vaccine design efforts. The high breadth (diversity in the number of variants that can be neutralized) and high potency of some bnAbs that neutralize HIV also makes them attractive candidates for immunotherapy.^6^, ^7^ Several bnAbs are currently under development as immunotherapeutics,^8^, ^9^, ^10^ which requires optimization of their biochemical properties for use as biologics. This process includes improvement of factors such as solubility and half-life, and selection against other features such as autoreactivity.^8^, ^9^, ^10^, ^11^ One feature that could play an important role is post-translational modification (PTM), due to its potential to substantially alter the biochemical landscape of a given protein.

One PTM that has been less frequently discussed in the context of therapeutic antibodies is cysteinylation, in which a cysteine residue in the antibody forms a disulfide with a free cysteine molecule.^12^ Some studies have shown that the presence of a cysteine modification in antibodies may interfere with engagement of their target, which would impede their application as therapeutic agents.^13^, ^14^ However, this PTM has not previously been reported in the context of broadly neutralizing antibodies to HIV.

The PCDN family is a lineage of antibodies isolated from an individual participant in the International AIDS Vaccine Initiative’s (IAVI) **Protocol C**, a longitudinal study of neutralization breadth in HIV-infected individuals.^5^ The donor in question, **PC76**, developed a broad serum response targeting the N332-glycan supersite (also known as the high-mannose patch), one of the key epitopes on Env, which involves *N*-linked glycans and amino-acid residues around the base of the hypervariable region 3 (V3) loop. In a previous study describing the neutralization properties of monoclonal antibodies (mAbs) from the PCDN lineage, our group determined crystal structures of two mAbs of intermediate breadth and potency, PCDN-27A and PCDN-27B.^15^ Notably, the heavy chain complementarity-determining region 3 (CDRH3) loops of the antibodies displayed an extended β-hairpin conformation, a common feature of anti-HIV bnAbs that recognize glycans. A total of 13 antibodies were characterized in this study that consisted of 12 mAbs and the unmutated common ancestor (UCA) of the lineage. All thirteen of these antibodies have a cysteine (Cys) residue at position 100 k, which is located at the base of CDRH3. Crystal structures confirmed that this residue is not involved in any intra- or inter-protein disulfide bonds, making it a candidate for cysteinylation. In this study, we show that this residue is indeed a site for cysteinylation as indicated by structural and biochemical techniques, and we present the first structure of a natively cysteinylated antibody.

## Results

### Heterogeneous expression of PCDN mAbs

**Figure 1:**
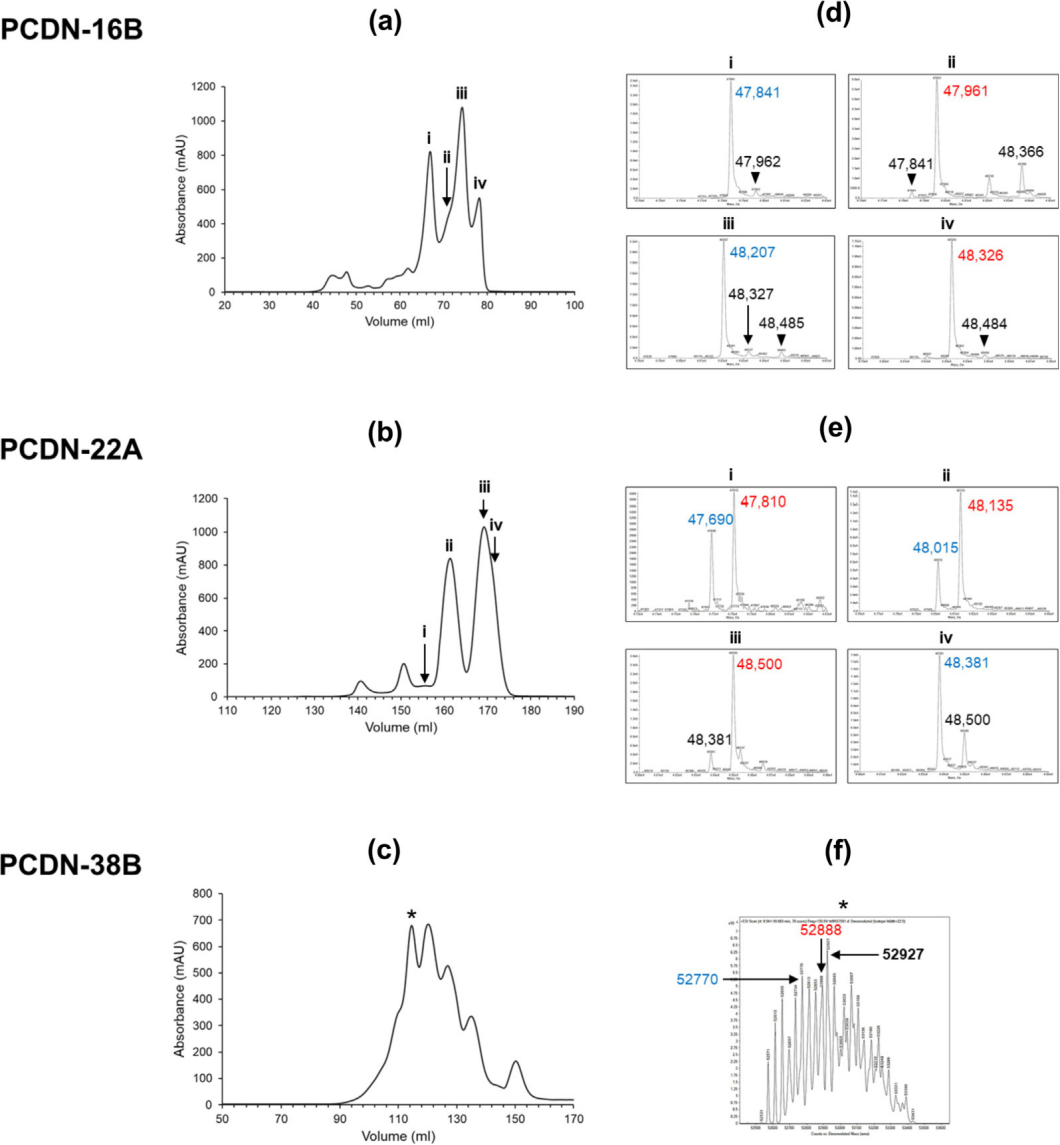
Heterogeneous profiles of mammalian cell-expressed PCDN antibodies during purification by cation exchange chromatography and electrospray ionization mass spectrometry (ESI-MS). Ion exchange chromatograms for (a) PCDN-16B, (b) PCDN-22A, and (c) PCDN-38B. Subpopulations of interest are indicated with numerals or an asterisk, and the respective mass spectrum for each population shown in (d) PCDN-16B, (e) PCDN-22A, and (f) PCDN-38B. Masses of prominent species are listed for clarity. Colored text within or between panels indicates a pair of species separated by 119 ± 1 Da with lighter members of each pair in blue and heavier species in red.

To investigate the properties of the PCDN antibodies, we recombinantly expressed each antibody as a fragment antigen-binding domain (Fab) in HEK293F cell culture. The purification of these Fabs, which involves a cation exchange chromatography step, revealed that these antibodies eluted as a collection of multiple subpopulations (Figure 1(a–c)). Such heterogeneity has been known to occur due to formation of antibody light chain (LC) dimers as an unintended product of recombinant Fab expression. However, even after using polyacrylamide gel electrophoresis (PAGE) to identify potential LC dimer populations, the population corresponding to properly assembled Fab still exhibited a heterogeneous profile.

To further resolve the differences between these subpopulations, we subjected samples of the Fab species to electrospray ionization mass spectrometry (ESI-MS) at The Scripps Research Institute’s Center for Metabolomics and Mass Spectrometry. The ESI-MS results confirmed that the identified species did indeed differ by mass (Figure 1(d, e)). We can attribute most of the variation in mass to differential cleavage of the signal peptide of the LC constructs; species could be characterized with up to four residues from the signal peptide uncleaved at the LC N terminus, and those with the signal peptide completely cleaved, but with the first four residues of the light chain variable region (V_L_) also deleted (Table 1). Certain Fabs also appear to have additional post-translational modifications in the form of *N*-linked glycosylation, which leads to extensive heterogeneity in their ion exchange chromatograms (Figure 1(c, f)).

**Table 1:** Sequence variations and post-translational modifications observed in the PCDN antibodies in this study.

	Mass shift (Da)	Modification	Chain
α	−129	Glutamic acid loss	LC
β	−113	Leucine/isoleucine loss	LC
γ	−99	Valine loss	LC
δ	−17	Pyroglutamic acid formation (Gln)	HC
ε	−2	Disulfide bond formation	Both
ζ	+57	Glycine addition	LC
η	+99	Valine addition	LC
θ	+101	Threonine addition	LC
ι	+119	Cysteinylation	HC
κ	+137	Histidine addition	LC

In addition to these modifications, we observe mass spectra species that can be distinguished by differences in mass of 118–120 Da. In PCDN-16B, the four most abundant species can be grouped into two pairs with respective masses 47,841 and 47,961 (difference: 120 Da; Figure 1(d), populations i and ii), and 48,207 and 48,326 (difference: 119 Da; Figure 1(d), populations i and ii). In PCDN-22A, such mass differences are again observed, but in a slightly more complex pattern: the major mass peaks for the respective four subpopulations were 47,810, 48,135, 48,500, and 48,381 Da, respectively (Figure 1(e)). Of these, only the latter two are separated from each other by 119 Da (Figure 1(e), populations iii and iv). The former two do not, but their respective mass spectra show minor peaks of mass 47,690 and 48,015 Da (populations i and ii). Nonetheless, both spectra preserve the aforementioned pattern, as these minor-major peaks represent mass pairs 47,690/47,810 Da and 48,015/48,135 Da, respectively. Each of these pairs features a separation of 120 Da.

PCDN-38B is yet another antibody for which we observe this variation, but with a much more complex pattern than the other two mAbs. Mass spectra of species from the ion exchange chromatogram of this variant were difficult to interpret due to the presence of two potential *N*-linked glycosylation sites (^H^Asn26 and ^L^Asn70) within the Fab domain. However, one spectrum showed two minor peaks of respective masses 52,770 and 52,888 Da (a mass disparity of 118 Da; Figure 1(c, f)). Referring to the Delta Mass database of the Association of Biomolecular Research Facilities (https://abrf.org/delta-mass, accessed January 7, 2021), these mass shifts of 119 ± 1 Da can be attributed to a number of chemical modifications, one of which is cysteinylation.

### Crystal structures of PCDN mAbs confirm cysteinylation of Cys100k

Due to the close overlaps of species from the ion exchange stage of purification, we sought to better separate them in advance of crystal trials, as reduction of heterogeneity can facilitate crystallization and increase confidence in the interpretation of resulting structural models. For PCDN-16B and 22A, we therefore re-purified a sub-component of the most abundant species from the ion exchange step, using a more focused gradient and slower elution rate. The purification method for PCDN-38B already employed a focused gradient and no further optimization was required prior to crystal trials.

**Figure 2:**
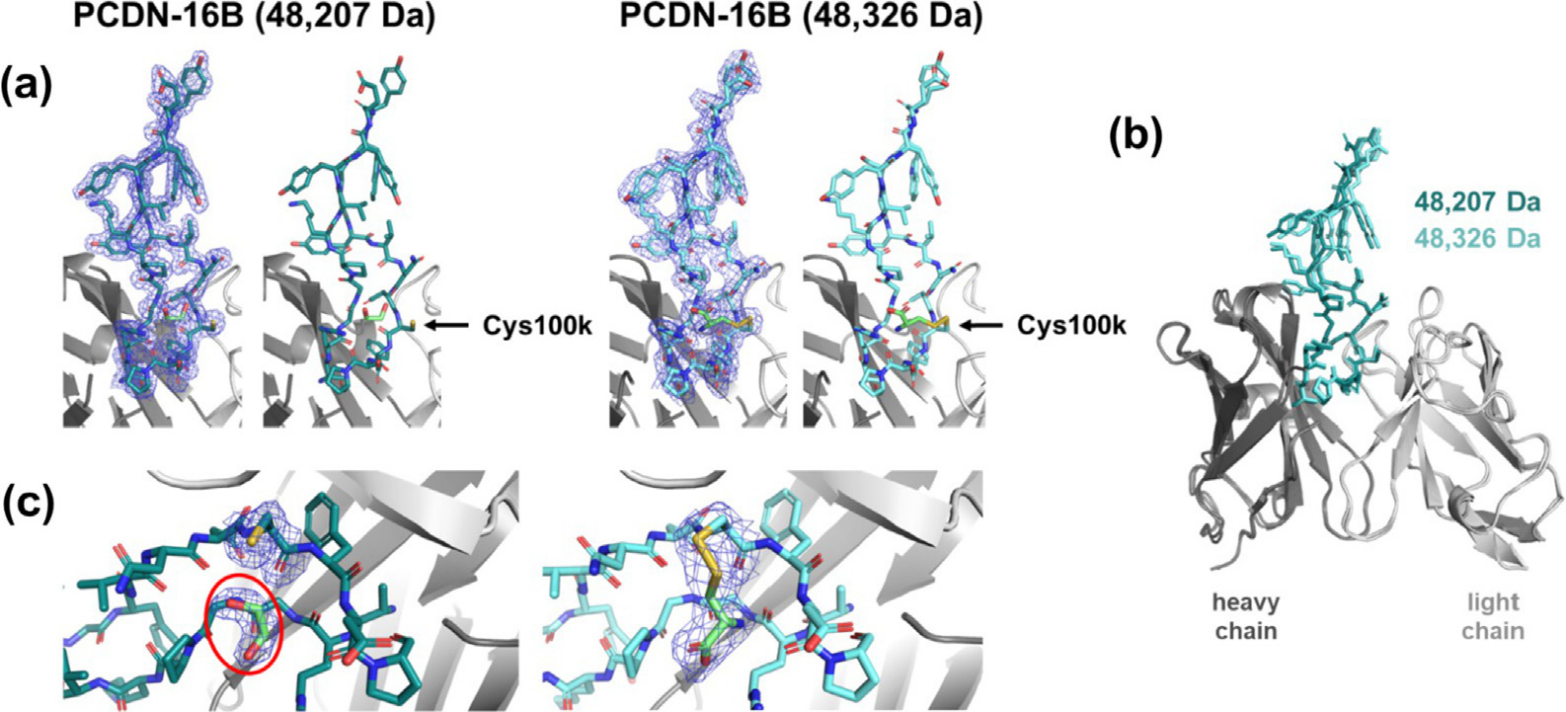
Crystal structures of both PCDN-16B species demonstrate that cysteinylation of the CDRH3 is the cause of the gain in mass. (a) Near-atomic models of the respective CDRH3 loops, shown with and without electron density maps. (b) Structural alignment of both forms of the antibody shows that the PTM does not affect the conformation of the loop. (c) Close-up views of electron density at residue Cys100k on both structures. The 48,207-Da species is colored in teal, and the 48,326-Da species in aquamarine. A bound ethylene glycol molecule is shown within the red oval. 2F_o_-F_c_ maps are contoured at 1.0σ.

**Figure 3:**
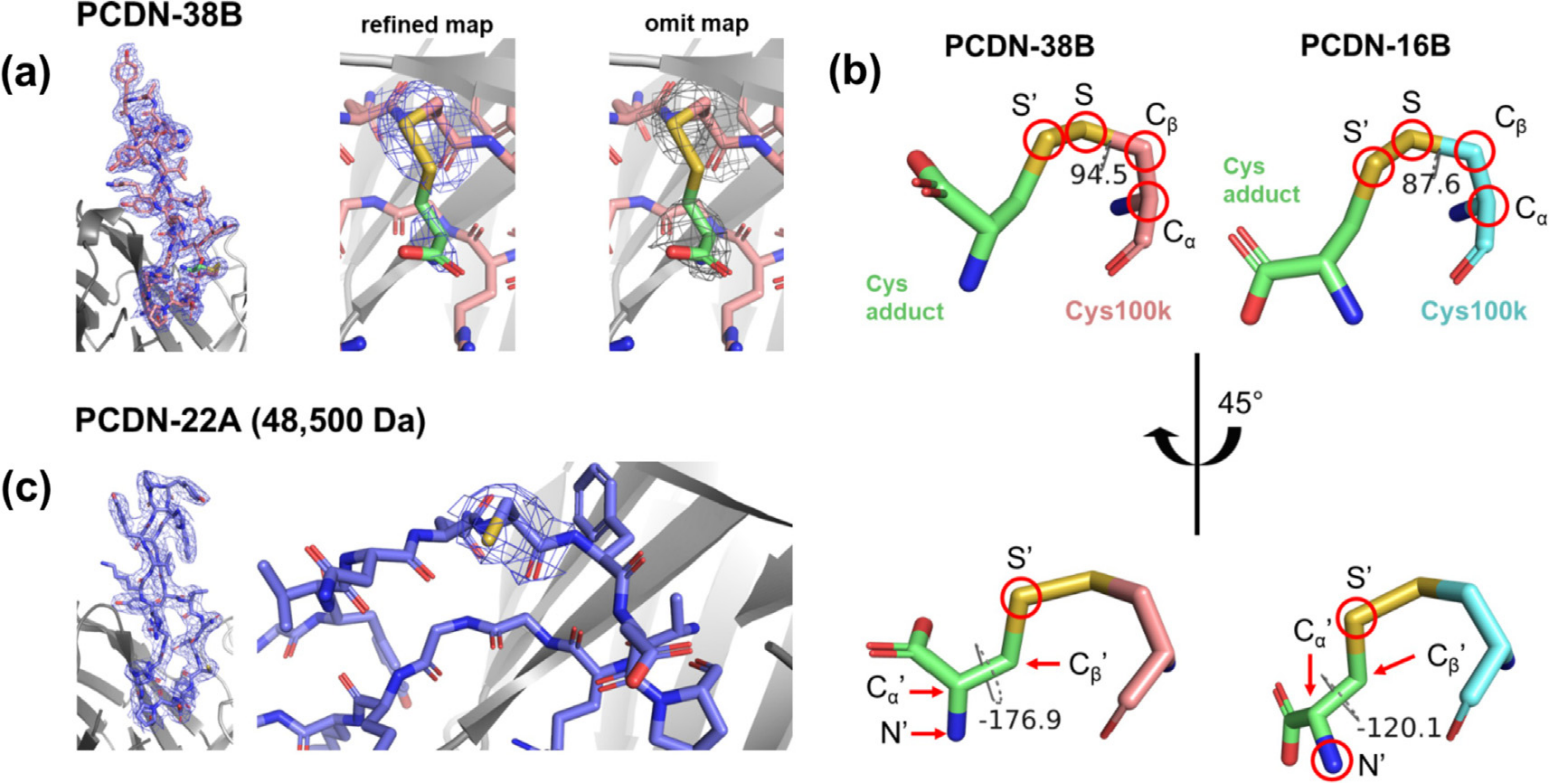
Crystal structures confirm cysteinylation of PCDN-38B, whereas for PCDN-22A, it is inconclusive. (a) Electron density maps of the entire CDRH3 and Cys100k of PCDN-38B. (b) Comparison of dihedral angles in PCDN-38B cystine to that of PCDN-16B. Red circles indicate atoms selected to highlight conformational similarities and differences between the respective cystines. (c) Electron density maps of CDRH3 of PCDN-22A, and of Cys100k specifically. 2F_o_-F_c_ maps are contoured at 1.0σ.

The resulting crystal structures of Fabs PCDN-16B and 22A (Table 2) showed that their variable regions (Fv), including CDRH3, resemble the previously published structures of PCDN-27A and PCDN-27B.^15^ In each case, the CDRH3 loop projects away from the center of mass of the Fab domain in an extended β-hairpin structure (Figure 2, Figure 3). However, each CDRH3 conformation is distinct. Two crystal structures were obtained for PCDN-16B subpopulations of mass 48,207 and 48,326 Da. Both show well-ordered electron density, allowing for comparison of their CDRH3 structures (Figure 2). The lighter species shows no modification at Cys100k where the closest non-protein entity is a molecule of ethylene glycol (Figure 2(a, c)). However, the other species, at 48,326 Da, displayed additional electron density emanating from the thiol group of Cys100k and was much more extensive than the density characteristic of a sulfhydryl group. Modeling of a free cysteine molecule into this density indicated formation of a disulfide bond with Cys100k (Figure 2(c)). The free amino and carboxylate groups were oriented in such a way that no obvious clashes or geometric restraint violations were observed (Figure 2(c)). We also noted that, despite the presence of the cysteine adduct, no significant differences were observed between the structures of the 48,326-Da and 48,207-Da species, either in the Fv (Cα root-mean-square deviation, RMSD, of 0.38 Å for 214 residues, where 1 Å = 0.1 nm) or in CDRH3 itself (Cα RMSD of 0.28 Å for 22 residues; Figure 2(b)).

**Table 2:** Data collection and refinement statistics for the PCDN antibody crystal structures.

Data collection	PCDN-16B (48,207 Da)	PCDN-16B (48,326 Da)	PCDN-22A (48,500 Da)	PCDN-38B
Beamline	APS 23-ID-D	APS 23-ID-D	SSRL 12–2	APS 23-ID-B
Wavelength (Å)	1.03320	1.03320	0.97946	1.03309
Space group	P2_1_	P2_1_	P2_1_2_1_2_1_	C2
Unit cell parameters (Å)	a = 46.8 b = 76.8 c = 62.4	a = 42.2 b = 115.2 c = 44.1	a = 70.1 b = 105.5 c = 145.9	a = 116.7 b = 68.1 c = 77.9
	β = 92.0°	β = 99.1°		β = 96.6°
Fab per ASU	1	1	2	1
Resolution (Å)*	50.0–1.93 (1.96–1.93)	50.0–2.46 (2.54–2.46)	50.0–2.70 (2.75–2.70)	50.0–2.08 (2.20–2.08)
Total reflections	88,907	32,886	176,789	128,368
Unique reflections*	32,727 (1,649)	14,022 (659)	29,990 (1,464)	36,353 (5,694)
Redundancy*	2.7 (2.7)	2.3 (2.2)	5.9 (6.0)	3.5 (3.6)
Completeness (%)*	98.7 (98.6)	94.0 (92.3)	99.3 (99.8)	99.1 (96.8)
<I/σ>*	7.4 (1.7)	7.1 (1.6)	9.0 (1.5)	9.0 (1.2)
R_sym_*	0.14 (0.70)	0.12 (0.52)	0.15 (1.59)	0.10 (0.98)
R_pim_*	0.10 (0.50)	0.09 (0.41)	0.07 (0.70)	0.06 (0.74)
CC_1/2_*	0.80 (0.43)	0.83 (0.48)	0.91 (0.51)	1.00 (0.70)

**Refinement statistics**				
Resolution (Å)	48.4–1.93	43.6–2.46	45.6–2.71	45.5–2.08
Reflections (work)	31,065	13,301	28,417	34,426
Reflections (test)	1,640	702	1,502	1,878
R_cryst_ (%)*	17.0 (25.6)	20.8 (31.3)	23.0 (39.2)	19.1 (30.1)
R_free_ (%)*	21.0 (32.6)	25.3 (35.3)	25.8 (41.7)	21.9 (34.0)
Number of atoms				
Protein	3,408	3,379	6,645	3,459
Glycans				74
Cysteine ligand		7		7
Solvent	562	75	9	259
Other ligands	35	19	16	69
Average B-value (Å^2^)				
All proteins	24	43	105	41
Fab 1	24	43	101	41
Fab 2			109	
Glycans				75
Cysteine ligand		48		70
Solvent	32	33	78	45
Other ligands	34	58	121	59
Wilson B-value (Å^2^)	22	37	72	36

**RMSD from ideal geometry**				
Bond length (Å)	0.003	0.002	0.002	0.003
Bond angles (°)	0.68	0.52	0.58	0.58

**Ramachandran statistics (%)**				
Favored	96.3	95.2	96.3	96.8
Outliers	0.0	0.2	0.0	0.2

PDB code	7RDJ	7RDK	7RDL	7RDM

* Values in parentheses refer to the highest resolution shell.

The crystal structure of PCDN-38B also showed evidence for cysteinylation (Figure 3(a)). The cystine of this antibody, however, displays weaker electron density than observed in PCDN-16B that may partially result from multiple alternate conformations exhibited by the cysteine adduct here. Ideally, alternate conformations would be modeled in the structure with each distinct orientation having an occupancy under 100%, proportional to the frequency of occurrence in the crystal. However, we found this region of the model to be unamenable to multiple conformations: alternate conformations of the cysteine adduct exhibited geometry violations and/or did not fit with the electron density maps. As such, we addressed this problem by calculating an unbiased, composite omit map (Figure 3(a), right panel) to remove phase bias associated with the various conformations of the adduct that we sampled. Based on the electron density that was observed in the omit map, we determined the cystine conformation with highest occupancy, and modeled it into the crystal structure (Figure 3(a)). Optimum definition of the refined electron density was found at around 60% occupancy of the adduct, but we decided not to model any other alternate conformations due to low confidence in their positions.

**Figure 4:**
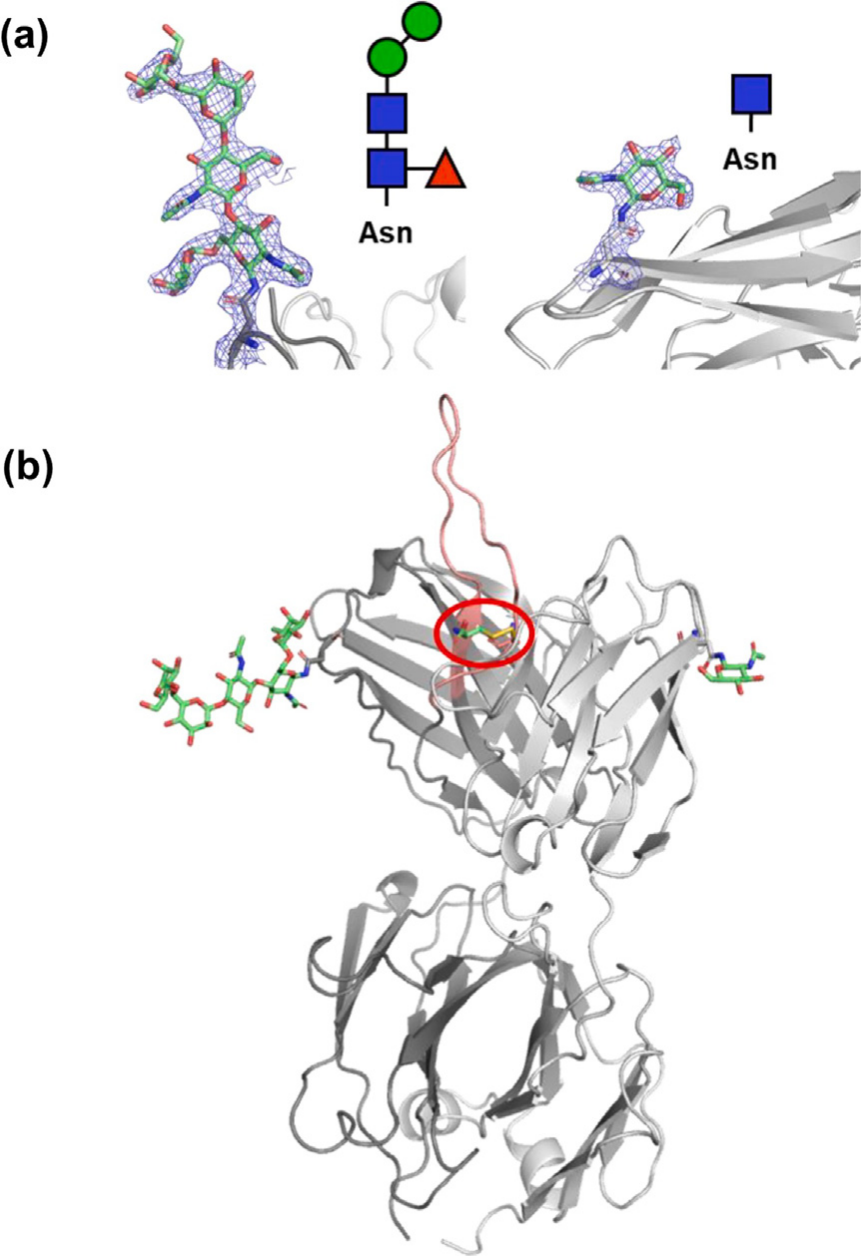
*N*-linked glycosylation in the PCDN-38B Fab. (a) Electron density maps of the N-linked glycans attached to ^H^Asn26 (N26, left) and ^L^Asn70 (N70, right). Chemical identities of the carbohydrate residues are also indicated, where blue squares = N-acetylglucosamine, green circles = mannose, and red triangles = fucose. 2F_o_-F_c_ maps are contoured at 1.0σ. (b) Structural overview of post-translational modifications observed on the PCDN-38B Fab. The location of the modified cysteine is encircled in red on CDRH3, which is in pink.

For the most occupied cystine conformation in PCDN-38B, the disulfide bond formed to Cys100k adopts a very similar conformation to the cystine of PCDN-16B as shown by the dihedral angles between atoms Cα—Cβ—S—S’, where S’ represents the sulfur of the adduct (Figure 3(b), upper panel). The orientation of the adduct is superficially similar in both Fabs, with the free amino group in each facing the β-strands of the CDRH3 while the carboxylate group is directed away from the protein. However, the dihedral angles over the S’—Cβ’—Cα’—N’ atoms (all on the cysteine adduct) indicate an overall rotation of the acetamido group by approximately 57° in the two structures (Figure 3(b)). In addition to electron density corresponding to the cysteine adduct, we also see some ordered density for both expected *N*-linked glycans in Fab 38B (Figure 4).

In contrast, the structural data did not definitively confirm cysteinylation on PCDN-22A. The crystal structure from the 48,500-Da species, which would be expected to be the cysteinylated counterpart of the 48,381-Da isoform that we solved earlier, showed no electron density corresponding to an adduct on Cys100k (Figure 3(c)). Thus, we will not discuss it further in this study.

### Cysteinylation of PCDN-16B has no detrimental effect on binding to its antigenic target

To interrogate the possibility of cysteinylation in CDRH3 affecting how PCDN antibodies interact with the Env trimer, we performed a series of biolayer interferometry (BLI) experiments. We selected PCDN-16B for this investigation, as not only does it show strong evidence for cysteinylation, but we also can separate cysteinylated from non-cysteinylated fractions to some extent. The binding target was BG505 SOSIP, a soluble construct of the Env trimer that is especially amenable to structural and biochemical investigations.^16^

**Figure 5:**
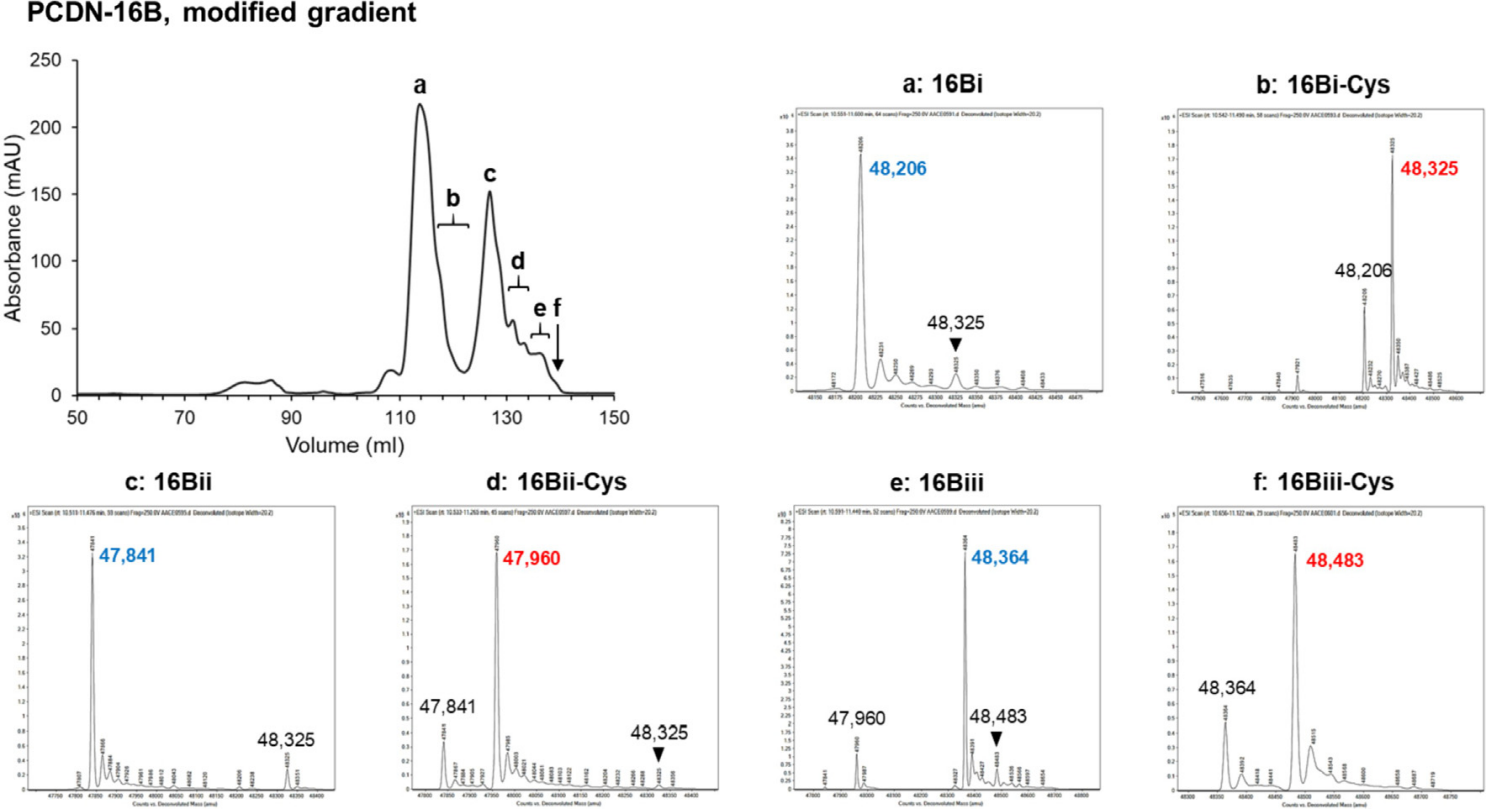
Cation exchange chromatogram of PCDN-16 using a focused gradient, alongside ESI-MS results of the respective subpopulations. Each population is indicated with a letter, and respective mass spectra are shown. Masses of prominent species are listed for clarity, and bold type indicates major constituents. Colored text additionally indicates a pair of species separated by 119 ± 1 Da in the adjacent panels (a/b; c/d; e/f). Lighter members of each pair are indicated in blue, and heavier species in red.

To this end, we purified a new expression lot of PCDN-16B Fab. During purification of the protein, we identified six key subpopulations with unique masses, as confirmed by ESI-MS (Figure 5). The relationship between these species retained the patterns described earlier, namely: six populations represent three pairs of 16B isoforms, each pair is characterized by a distinct light chain N terminus, and each member of the pair differs by cysteinylation status (Table 3). The pairs were named 16Bi, 16Bii, and 16Biii, respectively. The four masses corresponding to pairs 16Bi and 16Bii were observed in the previous expression lot as major peaks (16Bi: 48,206 and 48,325 Da; 16Bii: 47,841 and 47,960 Da; Figure 1(d)) within the respective mass spectra (allowing for an uncertainty of ± 2 Da), while the masses of 16Biii (48,364 and 48,483 Da) were present as minor peaks (Figure 1(d), populations ii–iv).

**Table 3:** Summary of the six most prominent subpopulations of PCDN-16B.

16B LC N terminus	Mass (Da)	Shift from calculated (Da)	Alternate name
Signal peptide: VH	48,206	+209	16Bi
	48,325	+328	16Bi-Cys

E1 deletion	47,841	−156	16Bii
	47,960	−37	16Bii-Cys

Signal peptide: TGVH	48,364	+367	16Biii
	48,483	+486	16Biii-Cys

**Figure 6:**
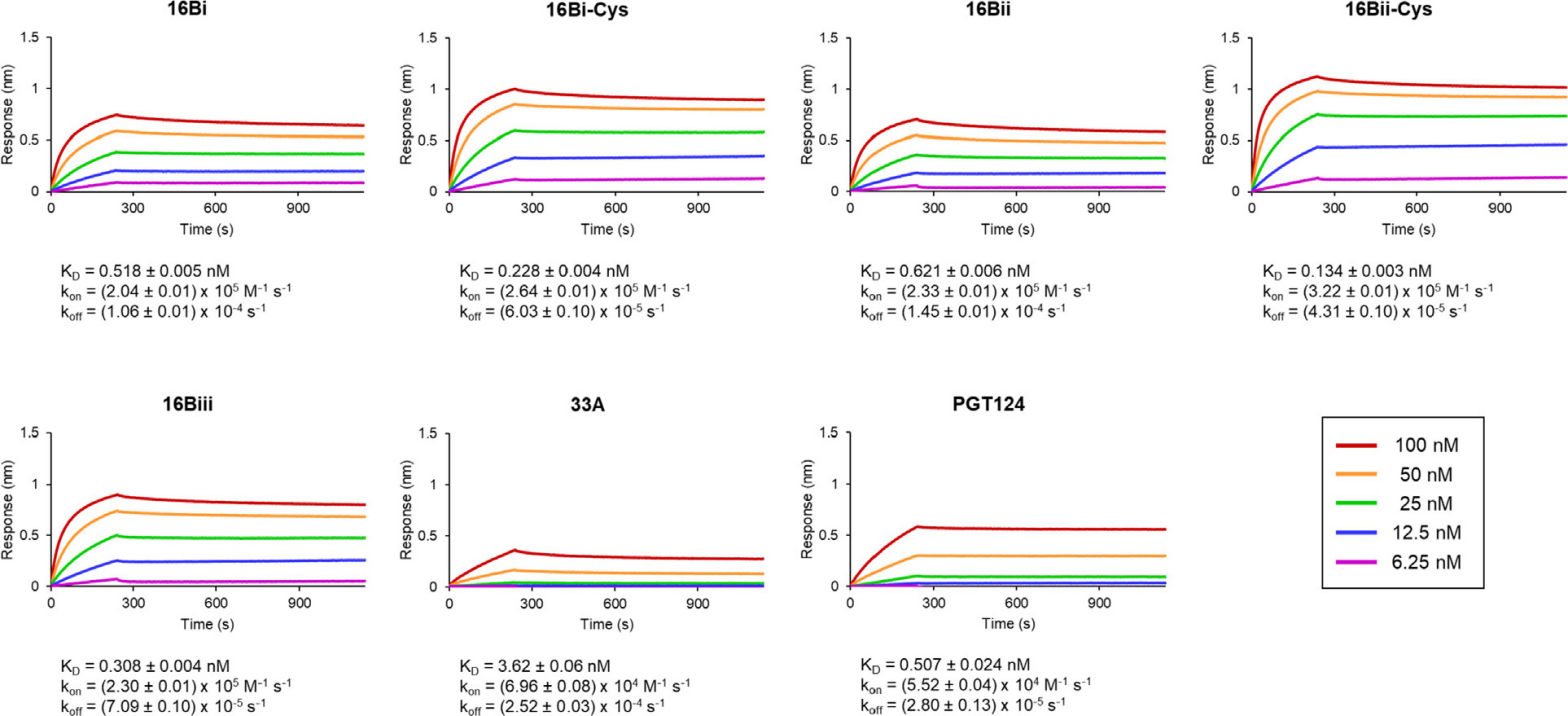
Biolayer interferometry of interactions between PCDN-16B isoforms (Fabs) and BG505 SOSIP show that cysteinylation is associated with modest binding enhancement. Data were collected at five concentrations of the antigen construct, with each measurement colored as shown in the legend. The mature PCDN antibody 33A as well as PGT124 were also examined for comparison.

**Figure 7:**
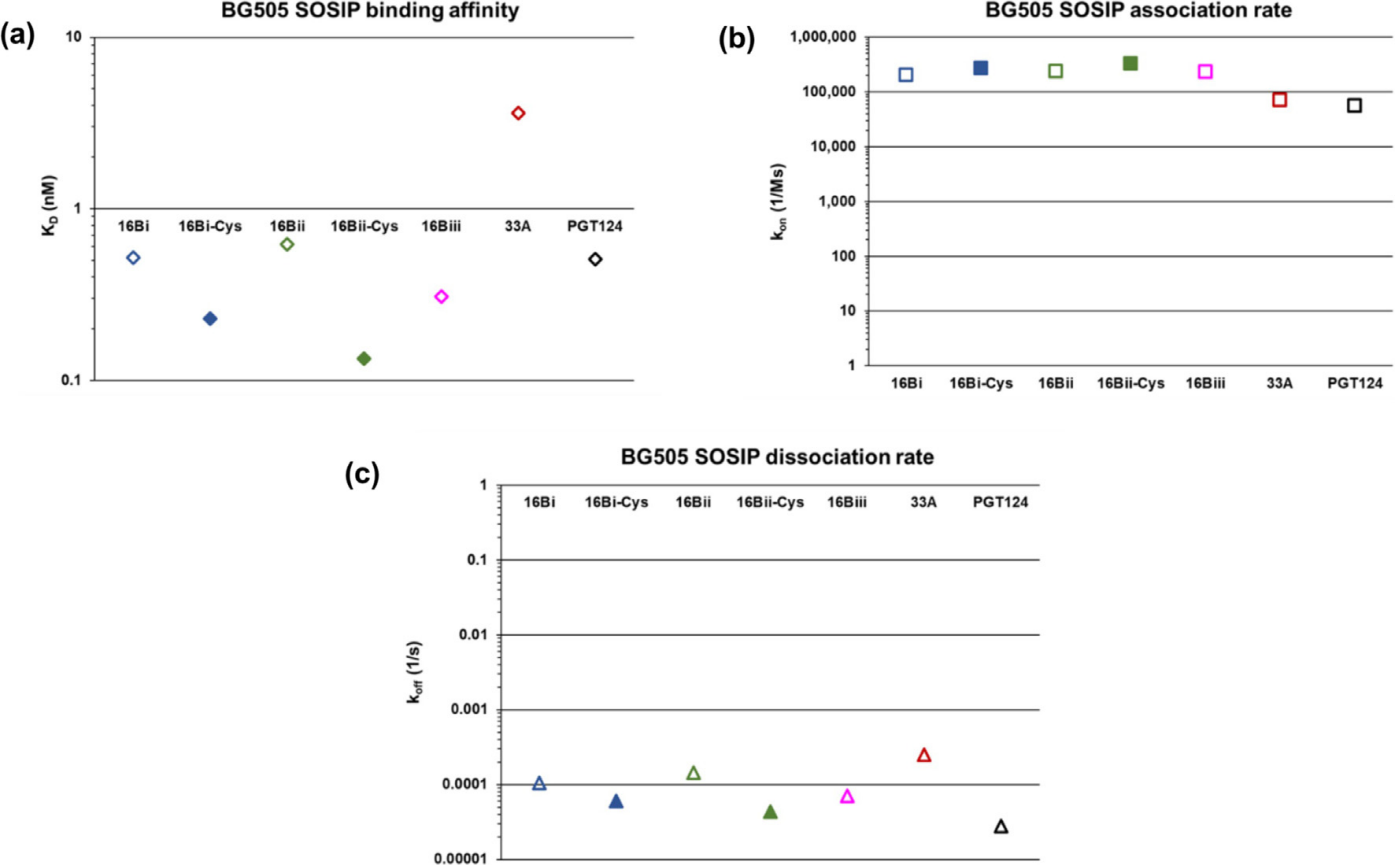
Graphical depiction of binding parameters obtained via biolayer interferometry (BLI) experiments. (a) Binding affinity (K_D_), (b) association rates, and (c) dissociation rates of antibody Fab:BG505 SOSIP complexes.

In these experiments, we assessed the association and dissociation rates of five of the six possible 16B:BG505 SOSIP Env complexes, including as references PCDN-33A (a mature member of the PCDN lineage) and PGT124, an N332-glycan supersite-targeting bnAb from a different lineage.^15^, ^17^ We were unable to examine binding of the cysteinylated 16Biii isoform (48,483 Da) due to insufficient amounts of protein. The results showed that all tested versions of PCDN-16B bound to the BG505 construct with K_D_ values ranging from 0.1 to 0.6 nM (Figure 6, Figure 7(a)). PCDN-33A and PGT124 bound with K_D_ values of 3.62 and 0.51 nM, respectively (Figure 6, Figure 7(a)), indicating comparable binding to the 16B isoforms. The changes in K_D_ values between non-cysteinylated and cysteinylated forms of 16B show that binding affinity increases slightly when the Fab is modified, indicating that the PTM does not negatively impact its function. For 16Bii, for example, the K_D_ value decreases 4.6-fold (Figure 6, Figure 7(a)).

The association rates show a similar positive effect of cysteinylation on binding, but less pronounced. In this case, we obtained values for non-cysteinylated 16B isoforms close to 2 × 10^5^ M^−1^ s^−1^, which increased very slightly when the isoforms were modified (Figure 6, Figure 7(b)). All were moderately higher than the respective association rates of 33A and PGT124. Thus, the results indicate slightly faster association of 16B with antigen compared to 33A and PGT124, as well as a marginally favorable effect of Cys100k cysteinylation on antibody-antigen interaction (Figure 6, Figure 7(b)). Dissociation rates of 16B to BG505 followed a similar trend where k_off_ values slightly decreased when each 16B isoform was cysteinylated (Figure 6, Figure 7(c)). Again, this effect was slightly more pronounced for 16Bii over the 16Bi isoform pair. The mature antibody PCDN-33A was within this range of the 16B dissociation rates, whereas PGT124 has slightly slower k_off_ compared to the best 16B isoform (16Bii-Cys) (Figure 6, Figure 7(c)). Thus, Fab 16B dissociates from Env at a comparable rate to the closely related 33A, and the presence of the PTM slows down this process, essentially extending the time for which antigen is bound. As such, none of the binding parameters indicates diminished binding of PCDN-16B due to cysteinylation, but rather this modification effects a modest but measurable improvement on binding.

### Prior characterization of cysteinylation in antibodies

Prior to the discovery of cysteinylation in the PCDN lineage, this PTM had been described in a few antibodies. In these instances, we could only identify one antibody in which the CDRH3 was the location of the modification: MAB007, for which no structural model has been published.^12^, ^13^ To confirm that there were indeed no published structures of any other natively cysteinylated antibodies, we searched the Protein Data Bank (PDB; rcsb.org) for structures in which cysteine molecules occurred as ligands, covalently or noncovalently bound.^18^, ^19^ We found 126 such structures (as of June 2021), of which only two were potentially cysteinylated antibodies. The first of these was the crystal structure of 412d, an anti-HIV CD4-induced antibody (PDB ID: 1RZG).^20^ The structural model contained two Fab molecules, and the C-terminal residue of the light chain (Cys214) on one of these antibodies formed a disulfide bond with a seemingly unidentified dipeptide (sequence: Cys-Asp). However, this dipeptide most likely originates from the disordered C terminus of the Fab heavy chain (HC). The second was the fragment-crystallizable (Fc) domain of an antibody, the C_H_2 segment of which contains an insertion mutation of a cysteine residue for formation of an antibody-drug conjugate at residue S239, which was shown to be cysteinylated (PDB ID: 6P6D) or modified with maleimide-PEG8.^21^

To account for the possibility of antibody structures with potentially cysteinylated CDRH3 having been uploaded to the PDB without modeling of the cysteine adduct, we performed another search. Noting that Cys100k in the PCDN antibodies occurs within the amino-acid motif NCFD, the same sequon modified in MAB007, we used the National Center for Biotechnology Information’s (NCBI) BLAST tool (https://blast.ncbi.nlm.nih.gov/Blast.cgi) to search the PDB for protein structures for similar motifs. Within the top 100 results of this search, only two antibodies were found: PCDN-27A and PCDN-27B, which are other members of the PCDN lineage reported in our 2016 study.^15^ It is worthwhile noting that the electron density maps for these structures showed no evidence for cysteinylation of Cys100k (PDB IDs: 5BZD, 5BZW).^15^

We acknowledge the low likelihood of specifically locating antibodies within the repertoire of all known protein structures (almost 200,000 as of June 2021) using a motif of only four residues in length. Accordingly, we searched the PDB for structures with sequences similar to CDRH3 in MAB007, as reported in Gadgil et al.^12^

**Figure 8:**
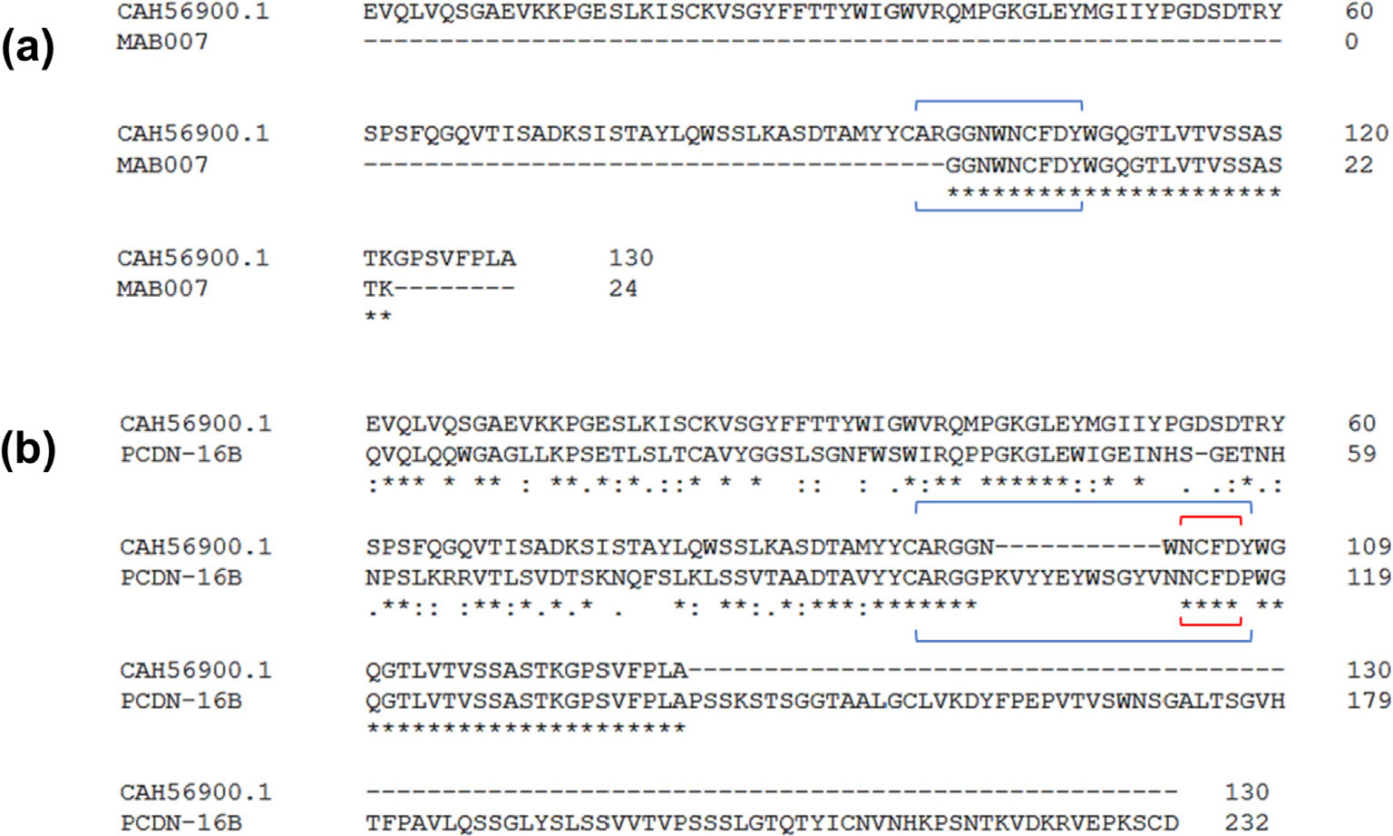
Comparison of cysteinylated antibodies MAB007 and PCDN-16B. (a) Alignment of a MAB007 HC peptide and an anti-IL15 antibody V_H_ demonstrates mutual identity in CDRH3, highlighting the CAH56900.1 sequence as the most probable representation of the MAB007 variable region. (b) Alignment of CAH56900.1 and 16B highlights the similarities in portions of their CDRH3 despite low similarity in other V_H_ subregions. Blue brackets delineate CDRH3 as defined in IMGT nomenclature, while red brackets emphasize the shared NCFD motif.

For this search, all of the top 100 results were antibodies, with three bearing intra-CDRH3 disulfide bonds. However, none of them had unpaired cysteines in this region. In a final attempt, we searched the PDB with the intact variable heavy chain sequence of MAB007. We could not find this sequence listed in any publications, leading us to first search an antibody database for similar sequences to CDRH3 of MAB007. We selected the abYsis database, where the antibody sequence repertoire is much larger than in the PDB (http://www.abysis.org/abysis/sequence_input/blast/blast_form.cgi). The top two results were identical antibody heavy chain variable region (V_H_) sequences under accessions CAH56900.1 and CAJ00024.1, each linked to a separate patent filed for antibody therapeutics to interleukin-15.^22^, ^23^ As this protein is reported to be molecular target of MAB007,^13^ we conclude that this is in fact the V_H_ of MAB007, or in the very least of a closely related variant (Figure 8). We performed a BLAST of this sequence in the PDB, which returned no antibodies with unpaired cysteines in CDRH3.

## Discussion

Cysteinylation in antibodies, while not widespread, poses an interesting challenge to therapeutic applications of antibodies due to its ability to potentially alter the normal function of these proteins. Generally, any form of heterogeneity constitutes an additional variable to be optimized when expressing proteins on an industrial scale. The PCDN antibody lineage exhibits heterogeneity due to native cysteinylation and other instances of differential processing. Considering that this lineage gave rise to multiple anti-HIV bnAbs of appreciable neutralizing breadth and potency, understanding the effects of these PTMs is beneficial to adaptation of these anti-HIV bnAbs for therapeutic use. While prior studies have predominantly used biochemical methods to characterize cysteinylation in antibodies,^12^, ^13^, ^14^ our study demonstrates the utility of structural biology techniques for precisely characterizing cysteinylation.

Notwithstanding, the importance of protein chromatography and mass spectrometry cannot be overstated for such analyses. The application of these techniques during purification of the PCDN antibodies drew our initial attention to the heterogeneity present and aided in characterization of the various underlying processes. Differential cleavage of the signal peptide of the PCDN light chain construct (sequence: MGWSCIILFLVATATGVH) accounts for most of the observed variation (Figure 1(d, e); Table 1). Three such LC variants are shared between PCDN-16B and 22A, which represent isoforms in increasing order of mass where: (1) the N-terminal glutamic acid residue (Glu1) is cleaved along with the signal peptide, (2) the two most C-terminal residues in the signal peptide (Val-His) are not cleaved, and (3) the number of uncleaved residues remaining from the signal peptide is four (Thr-Gly-Val-His). The PCDN-22A expression profile additionally shows evidence of a fourth isoform, in which the first four N-terminal residues of the light chain variable region (Glu-Ile-Val-Leu) are also excised along with the signal peptide. It is noteworthy that neither PCDN-16B nor 22A show any significant amount of Fab in which “proper” processing of the signal peptide is seen. Whether this pattern of variation is an artefact of signal peptide choice or cellular expression system is currently unknown, or whether this aberrant cleavage is reflected in the actual expression of these proteins in native B cells.

This diversity of isoforms is further complicated by cysteinylation, such that each LC-based variant exists in both cysteinylated and non-cysteinylated subpopulations. A curious observation is that for PCDN-16B, the cysteinylated species (shifted by +119 Da) elutes later during cation exchange chromatography, while the opposite occurs for 22A (Figure 1(a, b, d, e)). Considering that this method separates protein species on the basis of charge, such that increasing positive charge correlates with longer elution time, this finding indicates that cysteinylation of 16B appears to confer a greater positive charge, while the same modification somehow has the opposite effect on 22A. The reason for this may have to do with the initial charge of the CDRH3, where 22A has a higher net positive charge in this region than 16B and is then shifted by addition of a potentially zwitterionic adduct. As we only examined the effect of this PTM on 16B, whether this apparent shift in charge differentially affects the function of both antibodies is not known and would constitute an interesting future study.

Owing to the extreme heterogeneity observed in the biochemical profiles of PCDN-38B due to its glycosylation, none of the aforementioned patterns of LC sequence variation could be confidently interpreted. However, as the 38B constructs make use of the same signal peptide as 16B and 22A, it can be expected that similar isoforms exist for this antibody. It is important to mention that unlike in 16B and 22A in which a single peak clearly dominated the mass spectrum associated with each crystal structure, the mass spectrum for the 38B species was characterized by closely nested peaks of relatively similar abundance (Figure 1(f)). As such, we cannot say whether the crystal structure represents the 52,927-Da major peak, the 52,888-Da population (the putative cysteinylated version of the 52,770-Da peak), or some other species. Interestingly, the PCDN-16B Fab also has a potential *N*-linked glycosylation site at ^H^Asn52; however, the data show no indication that this modification occurs.

The crystal structures of PCDN-16B and 38B provide compelling evidence for cysteinylation. The structures of PCDN-16B were especially valuable as they represented two versions of the protein distinguished by 119 Da. The well-ordered density at Cys100k in the heavier species confirmed that cysteinylation, as opposed to any other PTM, is responsible for the shift in mass (Figures 1(d) and 2). Statistical measures of the crystallographic dataset for the 48,326-Da species are in agreement with this conclusion, specifically the B-values or temperature factors. These B-values are indicators of relative uncertainty of atomic positions within the model. In the crystal structure of 48,326-Da 16B, the average B-value of the cysteine adduct is 48 Å^2^, comparable to the protein portion of the model (43 Å^2^; Table 2). In the PCDN-38B crystal structure, the difference between B-values of the adduct and protein atoms is considerably larger (70 and 41 Å^2^, respectively); however, the average B-value of the *N*-linked glycans is 75 Å^2^, which is similar to that of the cysteine adduct. Also, we noted earlier that the electron density corresponding to the adduct in the 38B structure is less defined than in 16B, likely due to a series of alternate conformations for the ligand, most of which could not be easily modeled. The electron density then provides evidence of conformational disorder or heterogeneity, which would likely be associated with much higher B-values. Considering these points, the difference between B-values of protein and adduct in the 38B model may simply reflect the unique environment of the antibody or the crystal itself (Figure 3(a)).

The conformation adopted by the adduct in both antibodies is similar, with the dihedral angles Cα—Cβ—S—S’ differing by only 6.9° (Figure 3(b), upper panel). However, the dihedral angles S’—Cβ’—Cα’—N’ indicate that the relative locations of the amino and carboxylate groups of the adduct differ slightly more between antibodies (Figure 3(b), lower panel). Thus, the modified sidechains are either capable of free rotation and/or are differentially stabilized by unique interactions with the CDR loops of their respective antibodies. Considering the electron density and occupancy associated by the adduct in 38B, the former likely applies to its case. However, these differences could also be potentially related to the observation that cysteinylation has opposite effects on the charge of 16B and 22A.

Closer comparison of the Cys100k residue in both crystal structures of PCDN-16B shows that the lighter species (48,207 Da), while not having any density indicating a covalently bond adduct, does show evidence of a small chemical entity proximal to Cys100k. We modeled an ethylene glycol molecule into this density (Figure 2(a, c)), which, upon alignment with the 48,326-Da crystal structure, is superposed with the cysteine adduct of the latter structure. The density could also represent a low-occupancy cysteine in the 48,207-Da model. The corresponding mass spectrum does indicate some presence of the cysteinylated species in this population (Figure 1(d), population iii), further indicating that this Cys100k-proximal density in the 48,207-Da structure could represent a “less cysteinylated” population rather than a fully “non-cysteinylated” version of 16B. Should this in fact be the case, it would further demonstrate the sensitivity afforded by crystallography in informing on the relative amounts of cysteinylation in antibodies. However, this would require more definitive indication that this component of the model indeed corresponds to what was in the initial protein solution and may require further investigation.

The biophysical data show that cysteinylation does not impair binding of PCDN-16B to antigen. In fact, we observe slight improvements in all three examined binding parameters when the cysteine adduct is present (Figure 6, Figure 7). These findings contrast with previously described antibodies, such as mAb 001 and MAB007,^13^, ^14^ where their activity was diminished due to cysteinylation. Thus, the effect of cysteinylation on antibody function is not universally deleterious but can vary from antibody to antibody. Tolerance of cysteinylation by 16B relative to MAB007 could also be related to CDRH3 size. In PCDN antibodies, CDRH3 spans 22 residues, compared to 11 residues in MAB007 (Figure 8), following IMGT notation for this region (keeping consistency with MacLeod et al.).^15^ In Kabat nomenclature, the difference is 20 CDRH3 residues in the PCDN lineage versus 9 in MAB007. In either case, the PTM would produce a proportionally larger effect on CDRH3 of the latter antibody, including its volume. Alternatively, the lack of disruption of 16B function could result from the specific interactions of this antibody and Env, which we have yet to characterize. Considering that the site for cysteine addition is at the base of CDRH3, we may speculate that the adduct could create polar interactions (from the free amino and/or carboxylate groups) with the more exposed portions of the envelope trimer V3-glycan region such as the *N*-linked glycans at Asn301 and Asn332, or the GDIR motif, that are important for bnAb recognition of the N332-glycan supersite.^24^

We also observe that variation at the light chain N terminus has only mild effects on antigen binding affinity (Figure 6, Figure 7(a)). As with cysteinylation, there are very small shifts in K_D_ values that correlate with LC length: as the net number of residues at the N terminus increases in the non-cysteinylated isoforms, the K_D_ values decrease modestly. As such, non-cysteinylated 16Bii (47,841 Da), which lacks Glu1, is the least effective binder, while non-cysteinylated 16Biii (48,364 Da), retaining four residues from the signal peptide, is the strongest (Table 3; Figure 6, Figure 7(a)), albeit by very small margins. This observation is consistent, however, with the structure of the antibody where the LC N terminus is nestled on the face of the Fab bearing the complementarity-determining regions (CDRs). Additional residues at the normal N terminus could potentially improve interactions with antigen, either by increasing protein–protein interface interactions or through an allosteric effect on CDR and framework loops that are directly involved in binding Env. Interestingly, this effect is different when the Fab is cysteinylated: modified 16Bii exhibits slightly higher affinity than cysteinylated 16Bi (Figure 6, Figure 7(a)). It is not known why the combination of a shorter light chain and cysteinylation creates the best observed improvement of any binding parameter in this study (reduction of K_D_ by a factor of 4.6). As we could not test cysteinylated 16Biii under the BLI conditions in Figure 6, we do not know whether cysteinylation definitively reverses the trend seen for non-cysteinylated 16B LC variants. Interestingly, this pattern also holds true for the dissociation rates (Figure 6, Figure 7(c)).

Rates of association show no clear correlation between LC length and association rate (Figure 6, Figure 7(b)), especially for non-cysteinylated isoforms of 16B. Again, the lack of information on cysteinylated 16Biii prevents us from determining if shorter light chains indeed lead to increase in the rate of association to antigen. As the primary focus of this study is elucidation of the role of cysteinylation, further investigation of the effects of light chain variation is beyond the scope at this time.

Another noteworthy observation is that PCDN-16B shows higher binding affinity and association rate to antigen than PCDN-33A, as well as lower dissociation rates (Figure 7). This finding is unexpected since 16B is an early-stage antibody, isolated only after 16 months of infection, while 33A is more mature and was first observed in the donor’s serum after 33 months. Moreover, neutralization experiments in the study by MacLeod et al.^15^ confirm that PCDN-33A neutralizes Env with higher breadth and potency than 16B. However, one neutralization panel showed that PCDN-16B was able to neutralize one autologous virus variant that 33A could not, in 66 unique viral clones examined.^15^ This observation indicates that 16B is a more effective neutralizer than the more mature 33A for some strains, including BG505 SOSIP that we tested here. Another potential explanation for the increased effectivity of the less mature antibody involves the glycosylation profile of the antigen. The particular lot of BG505 SOSIP used in these binding studies was expressed in GnTI-deficient cell culture that produces high-mannose *N*-linked glycans, thereby eliminating complex and hybrid-type glycans which are normally also observed on Env. This shift from a wild-type glycosylation profile can alter interactions of antibodies to the V3-glycan region.^15,^^25^ Also, the effects of cysteinylation on 22A and 38B function were not tested and could behave differently in these antibodies from 16B.

The bioinformatic portion of our investigations was conducted across multiple databases and shows little evidence for any previously existing structural characterization of natively cysteinylated antibodies. The initial search on the PDB for proteins targeting cysteine ligands yielded over a hundred results, of which only three contained any immunoglobulin-related domains. One of these was an antibody Fab-leptin complex (PDB ID: 3V6O), for which the leptin, not the antibody, harbored the PTM.^26^ The other two consisted of an Fc domain and anti-HIV antibody Fab, respectively. The Fc was indeed cysteinylated at an unpaired cysteine residue (PDB ID: 6P6D); however, this amino acid was the result of an insertion mutation to alter the functionality of the domain and, hence, is not an instance of native cysteinylation.^21^ In Fab 412d, the C-terminal cysteine of the LC was bound to a cysteine from a dipeptide (PDB ID: 1RZG).^20^ The sequence of this dipeptide (Cys-Asp) matches residues in the HC C terminus to which the LC normally forms a disulfide bond, cross-linking both chains. Further inspection of the model shows that this region of the corresponding HC has not been modeled, likely due to disorder. Thus, PCDN-16B and 38B here constitute the first crystal structures of cysteinylated wild-type antibodies.

Why then has cysteinylation not been observed in antibody structures until now? Heavy-chain sequence alignment of PCDN-16B and MAB007 illustrates a shared four-residue motif (NCFD) in CDRH3 (Figure 8(b)) that is derived from the J_H_ gene. The PCDN lineage uses the J_H_5*01 allele^15^ and MAB007 originates from a similar, if not identical, J_H_ allele. Curiously, the J_H_5*01 germline sequence is NWFD, which is well-represented in CDRH3 sequences with highest identity to MAB007, as we observed from the sequence BLAST results. This putative Trp → Cys mutation (TGG to TGC or TGT) thus appears to be somewhat rare, which becomes more noteworthy considering that every PCDN mAb (UCA included) has a Cys at this position. Thus, a key functional role for this cysteine residue is suggested and established very early in the development of this anti-HIV lineage. We have shown, for PCDN-16B at present, that cysteinylation does not negatively impact but slightly improves binding. However, we did not investigate its effects on PCDN-22A and PCDN-38B. As such, future work may focus on testing these antibodies. Whether cysteinylation can affect *in vivo* antibody function is still not known, mostly due to the uncertainty regarding the question of when this PTM is added. There is some debate regarding whether antibodies are cysteinylated during processing in the endoplasmic reticulum (intracellular), or after secretion into the expression media (extracellular).^13^, ^27^, ^28^ The latter would indicate that this PTM is more of a biproduct of cell culture expression, but still highly relevant to the use of antibodies as therapeutics. Future work is thus required to address whether cysteinylation is involved in the mechanism of HIV-1 neutralization in this antibody lineage, which would require further methods development to cleanly separate cysteinylated versus non-cysteinylated IgG populations.

## Materials and methods

### Protein expression, purification steps

The antibodies were expressed as Fab in HEK293F cells with the FreeStyle 293 expression system (Invitrogen), transfecting HC and LC constructs in a 2:1 ratio. The Fabs were purified first by affinity chromatography (KappaSelect; GE Healthcare) and then by cation exchange chromatography (SP HP; GE Healthcare). For PCDN-16B and 22A, the ion exchange elution was initially conducted over a gradient of 0–50% elution buffer lasting 180 min, at a 0.8 ml/min flow rate. For improved resolution, a subset of the fractions was re-purified on a more focused gradient, 0–20% elution buffer for 240 min, at a 0.4 ml/min flow rate. The PCDN-16B lot re-expressed prior to biolayer interferometry was purified over 0–15% elution buffer, an even more focused gradient. PCDN-38B was purified over a 0–10% elution buffer gradient, with the other parameters corresponding to those used in the repurification of the other Fabs.

The antigen BG505 SOSIP was transiently expressed in HEK293S cells and harvested after 7 days of expression. The antigen was purified first via GN Lectin affinity chromatography, and then through size exclusion chromatography (Superdex 200 16/60; GE Healthcare).

Purified Fab fractions of interest (ones that corresponded to peak crests or distinct shoulders) were analyzed via polyacrylamide gel electrophoresis (PAGE), with each species run under both reducing and non-reducing conditions. Fab-containing samples were judged to be ones with bands at ∼50 kDa on the non-reducing lane, and at ∼25 kDa on the reducing lane. For Fab samples that were purified twice via cation exchange chromatography, PAGE was run on eluents from both runs.

### Mass spectrometry

Molecular weights of Fab fractions were determined by electrospray ionization mass spectrometry (ESI-MS) conducted at the Center for Metabolomics and Mass Spectrometry. Samples were run through an Agilent PLRP-S 100 Å 5 µm column, number 0006140735-10. A column guard was used to protect the column from overloading and from particulates. The autosampler used was an Agilent Technologies 1200 series autosampler, and the instrument an Agilent Technologies 6230 TOF LC/MS with a Dual AJS ESI ion source. The LC/MS gradient makes use of two solvents: Solvent A being 0.1% Formic Acid in H_2_O, and Solvent B being 0.1% Formic Acid in ACN. The gradient consisted of three steps: the first flowing 95% Solvent A and 5% B for 5 min, the second ramping up the concentration of B to 90% for 10 more min, and finally returning the concentration of B to 5%, flowing for another 1 min. The gradient was run at a flow of 300 µL/min at a pressure of 400 bar (40 MPa).

### Crystal Trials, Data Collection, Processing, refinement

Crystal trials were set up with Fabs initially in 20 mM sodium acetate (pH 5.6) buffer, except for PCDN-38B which was in a buffer consisting of 20 mM Tris (pH 7.4) and 150 mM NaCl. The 48,207-Da population of PCDN-16B crystallized at 7.95 mg/ml in conditions of 0.2 M disodium tartrate, 20% (w/v) PEG 3350. The 48,326-Da species crystallized at 5.87 mg/ml in 0.2 M lithium sulfate, 20% (w/v) PEG 3350. PCDN-22A (mass: 48,500 Da) initially produced crystals at 6.06 mg/ml in 0.1 M Tris (pH 7), 0.2 M magnesium chloride, 10% (w/v) PEG 8000. This set of conditions was further optimized to 0.1 M Tris (pH 7.37), 0.2 M magnesium chloride, 8% (w/v) PEG 8000. PCDN-38B (52,927 Da) crystallized at 12.6 mg/ml in 1.6 M sodium dihydrogen phosphate, 0.4 M dipotassium hydrogen phosphate, 0.1 M phosphate-citrate (pH 4.2). The crystals were flash-cooled by plunging into liquid nitrogen upon harvesting, and each one was cryoprotected using a mix of well solution and cryoprotectant: PCDN-16B (48,207 Da) in 10% ethylene glycol, 16B (48,326 Da) in two sequential solutions containing 30% glycerol and 10% ethylene glycol, respectively, 22A in 15% ethylene glycol, and 38B in 30% glycerol.

Data were collected at the Advanced Photon Source (APS) beamlines 23ID-D (both PCDN-16B isoforms) and 23ID-B (PCDN-38B), and at the Stanford Synchrotron Radiation Lightsource (SSRL) beamline 12-2 (PCDN-22A). Data processing was performed in HKL-2000^29^ for all datasets asides from PCDN-38B, which was processed using XDS.^30^ Molecular replacement was performed with Phaser,^31^ using as search model either a CDRH3 loop-truncated version of PCDN-27A, PDB ID: 5BZD (for non-cysteinylated PCDN-16B, PCDN-38B), or a similarly truncated version of PCDN-UCA (unpublished; used for cysteinylated PCDN-16B, PCDN-22A). Models were built in Coot,^32^ and PHENIX used for refinement and composite omit calculation.^33^ All structures were validated using MolProbity,^34^ and figures were rendered with PyMOL (PyMOL version 2.1.1; Schrödinger, LLC).

### Biolayer interferometry

Binding analysis of antibodies to BG505 SOSIP was conducted on an Octet RED96e (ForteBio, Fremont, CA) instrument. Fabs of the six PCDN-16B isoforms, PCDN-33A, and PGT124, were immobilized on Fab2G sensors (ForteBio, cat no 18-5125) previously hydrated in kinetics buffer (0.01% BSA, 0.002% Tween-20 in Dulbecco’s phosphate-buffered saline). Each Fab was loaded at a concentration of 10 μg/ml in buffer consisting of 20 mM Tris (pH 7.4) and 150 mM NaCl (TBS). The loaded sensors were dipped in SOSIP samples of varying concentrations in TBS: 100, 50, 25, 12.5, and 6.25 nM, respectively. Experiments were conducted with the following steps: (1) baseline in kinetics buffer for 60 s, (2) loading of Fab for 90 s, (3) shaking for 60 s, (4) a second baseline of 60 s, (5) association of SOSIP for 240 s, and (6) dissociation of the antigen into kinetics buffer for 900 s. A reference well containing just TBS was also run with each set of SOSIP samples, and was subtracted from the sample wells to correct for drift and buffer evaporation. All assays were performed at room temperature. Data were analyzed using the Octet RED Data Analysis software version 12.0.

### Database investigations

Investigation of the Protein Data Bank for occurrence of cysteine as ligands in protein structures started from the ligand page for L-cysteine (PDB ID: CYS, https://www.rcsb.org/ligand/CYS). We selected all search options asides from cases in which cysteines were incorporated in polymer sequences, and manually inspected all 126 hits for Fab, Fc and/or IgG domains. The NCBI BLAST of the **NCFD** motif was performed as a Protein BLAST, using this tetrapeptide as the query and limiting the target repertoire to the Protein Data Bank, keeping default parameters otherwise. The BLAST of the MAB007 CDRH3 used the same method, with its query the peptide (24 amino acids in length) given by Gadgil et al.^12^ This sequence was then used as a query in a BLAST on the AbYsis database, searching through all protein sequences accessible to the site. The single identical result was shared by two entries in the database (accessions CAH56900.1 and CAJ00024.1), which was used for the final BLAST on NCBI. Protein sequence alignments were performed in Clustal Omega.^35^

## Accession numbers

The crystal structures have submitted to the Protein Data Bank with the following accession codes: 7RDJ (non-cysteinylated PCDN-16B), 7RDK (cysteinylated PCDN-16B), 7RDL (PCDN-22A), and 7RDM (PCDN-38B).

## CRediT authorship contribution statement

**Oluwarotimi Omorodion:** Conceptualization, Methodology, Validation, Formal analysis, Investigation, Writing – original draft, Writing – review & editing, Visualization. **Ian A. Wilson:** Conceptualization, Methodology, Validation, Formal analysis, Resources, Supervision, Project administration, Funding acquisition, Writing – review & editing.

## Footnotes

1 **Abbreviations:**BLI, biolayer interferometry; bnAbs, broadly neutralizing antibodies; CDR(H3), complementarity-determining region (heavy chain 3); Env, envelope glycoprotein; ESI-MS, electrospray ionization mass spectrometry; Fab, fragment antigen-binding; Fc, fragment crystallizable; Fv, variable region; HC, heavy chain; IAVI, International AIDS Vaccine Initiative; LC, light chain; mAb, monoclonal antibody; NCBI, National Center for Biotechnology Information; PAGE, polyacrylamide gel electrophoresis; PDB, Protein Data Bank; PTM, post-translational modification; RMSD, root-mean-square deviation; UCA, unmutated common ancestor; V3; hypervariable region 3; V_H_, heavy chain variable region; V_L_, light chain variable region.

## References

[1] Burton D.R., Poignard P., Stanfield R.L., Wilson I.A. (2012). Broadly neutralizing antibodies present new prospects to counter highly antigenically diverse viruses. Science.

[2] Burton D.R., Hangartner L. (2016). Broadly neutralizing antibodies to HIV and their role in vaccine design. Annu. Rev. Immunol..

[3] Sok D., Burton D.R. (2018). Recent progress in broadly neutralizing antibodies to HIV. Nature Immunol..

[4] Doria-Rose N.A., Klein R.M., Daniels M.G., O'Dell S., Nason M., Lapedes A., Bhattacharya T., Migueles S.A. (2010). Breadth of human immunodeficiency virus-specific neutralizing activity in sera: clustering analysis and association with clinical variables. J. Virol..

[5] Landais E., Huang X., Havenar-Daughton C., Murrell B., Price M.A., Wickramasinghe L., Ramos A., Bian C.B. (2016). Broadly neutralizing antibody responses in a large longitudinal sub-Saharan HIV primary infection cohort. PLoS Pathog..

[6] Caskey M., Klein F., Nussenzweig M.C. (2019). Broadly neutralizing anti-HIV-1 monoclonal antibodies in the clinic. Nature Med..

[7] Walker L.M., Burton D.R. (2018). Passive immunotherapy of viral infections: “super-antibodies” enter the fray. Nature Rev. Immunol..

[8] Gaudinski M.R., Coates E.E., Houser K.V., Chen G.L., Yamshchikov G., Saunders J.G., Holman L.A., Gordon I. (2018). Safety and pharmacokinetics of the Fc-modified HIV-1 human monoclonal antibody VRC01LS: a phase 1 open-label clinical trial in healthy adults. PLoS Med..

[9] Sharma V.K., Misra B., McManus K.T., Avula S., Nellaiappan K., Caskey M., Horowitz J., Nussenzweig M.C. (2020). Characterization of co-formulated high-concentration broadly neutralizing anti-HIV-1 monoclonal antibodies for subcutaneous administration. Antibodies (Basel).

[10] Mahomed S., Garrett N., Karim Q.A., Zuma N.Y., Capparelli E., Baxter C., Gengiah T., Archary D. (2020). Assessing the safety and pharmacokinetics of the anti-HIV monoclonal antibody CAP256V2LS alone and in combination with VRC07-523LS and PGT121 in South African women: study protocol for the first-in-human CAPRISA 012B phase I clinical trial. BMJ Open.

[11] Kwon Y.D., Georgiev I.S., Ofek G., Zhang B., Asokan M., Bailer R.T., Bao A., Caruso W. (2016). Optimization of the solubility of HIV-1-neutralizing antibody 10E8 through somatic variation and structure-based design. J. Virol..

[12] Gadgil H.S., Bondarenko P.V., Pipes G.D., Dillon T.M., Banks D., Abel J., Kleemann G.R., Treuheit M.J. (2006). Identification of cysteinylation of a free cysteine in the Fab region of a recombinant monoclonal IgG1 antibody using Lys-C limited proteolysis coupled with LC/MS analysis. Anal. Biochem..

[13] Banks D.D., Gadgil H.S., Pipes G.D., Bondarenko P.V., Hobbs V., Scavezze J.L., Kim J., Jiang X.-R. (2008). Removal of cysteinylation from an unpaired sulfhydryl in the variable region of a recombinant monoclonal IgG1 antibody improves homogeneity, stability, and biological activity. J. Pharm. Sci..

[14] McSherry T., McSherry J., Ozaeta P., Longenecker K., Ramsay C., Fishpaugh J., Allen S. (2016). Cysteinylation of a monoclonal antibody leads to its inactivation. mAbs.

[15] MacLeod D.T., Choi N.M., Briney B., Garces F., Ver L.S., Landais E., Murrell B., Wrin T. (2016). Early antibody lineage diversification and independent limb maturation lead to broad HIV-1 neutralization targeting the Env high-mannose patch. Immunity.

[16] Sanders R.W., Derking R., Cupo A., Julien J.P., Yasmeen A., de Val N., Kim H.J., Blattner C. (2013). A next-generation cleaved, soluble HIV-1 Env trimer, BG505 SOSIP.664 gp140, expresses multiple epitopes for broadly neutralizing but not non-neutralizing antibodies. PLoS Pathog..

[17] Garces F., Sok D., Kong L., McBride R., Kim H.J., Saye-Francisco K.F., Julien J.-P., Hua Y. (2014). Structural evolution of glycan recognition by a family of potent HIV antibodies. Cell.

[18] Berman H.M., Westbrook J., Feng Z., Gilliland G., Bhat T.N., Weissig H., Shindyalov I.N., Bourne P.E. (2000). The Protein Data Bank. Nucleic Acids Res..

[19] Burley S.K., Bhikadiya C., Bi C., Bittrich S., Chen L., Crichlow G.V., Christie C.H., Dalenberg K. (2021). RCSB Protein Data Bank: powerful new tools for exploring 3D structures of biological macromolecules for basic and applied research and education in fundamental biology, biomedicine, biotechnology, bioengineering and energy sciences. Nucleic Acids Res..

[20] Huang C., Venturi M., Majeed S., Moore M.J., Phogat S., Zhang M., Dimitrov D.S., Hendrickson W.A. (2004). Structural basis of tyrosine sulfation and VH-gene usage in antibodies that recognize the HIV type 1 coreceptor-binding site on gp120. Proc. Natl. Acad. Sci. U. S. A..

[21] Gallagher D.T., McCullough C., Brinson R.G., Ahn J., Marino J.P., Dimasi N. (2019). Structure and dynamics of a site-specific labeled Fc fragment with altered effector functions. Pharmaceutics.

[22] J.G.J. van der Winkel, M.A. Van Dijk, J. Schuurman, A.F. Gerritsen, O. Baadsgaard, J. Petersen, (2004). WO2004076620A9.

[23] F. Beurskens, J. Schuurman, P. Parren, J. Petersen, O. Baadsgaard, (2005). WO2005044303A1.

[24] Kong L., Torrents De La Peña A., Deller M.C., Garces F., Sliepen K., Hua Y., Stanfield R.L. (2015). Complete epitopes for vaccine design derived from a crystal structure of the broadly neutralizing antibodies PGT128 and 8ANC195 in complex with an HIV-1 Env trimer. Acta Crystallogr. D.

[25] Garces F., Lee J.H., de Val N., Torrents de la Pena A., Kong L., Puchades C., Hua Y., Stanfield R.L. (2015). Affinity maturation of a potent family of HIV antibodies is primarily focused on accommodating or avoiding glycans. Immunity.

[26] Carpenter B., Hemsworth G.R., Wu Z., Maamra M., Strasburger C.J., Ross R.J., Artymiuk P.J. (2012). Structure of the human obesity receptor leptin-binding domain reveals the mechanism of leptin antagonism by a monoclonal antibody. Structure.

[27] Kita A., Ponniah G., Nowak C., Liu H. (2016). Characterization of cysteinylation and trisulfide bonds in a recombinant monoclonal antibody. Anal. Chem..

[28] Zhong X., He T., Prashad A.S., Wang W., Cohen J., Ferguson D., Tam A.S., Sousa E. (2017). Mechanistic understanding of the cysteine capping modifications of antibodies enables selective chemical engineering in live mammalian cells. J. Biotechnol..

[29] Otwinowski Z., Minor W. (1997). Processing of x-ray diffraction data collected in oscillation mode. Methods Enzymol..

[30] Kabsch W. (2010). XDS. Acta Crystallogr. D.

[31] McCoy A.J., Grosse-Kunstleve R.W., Adams P.D., Winn M.D., Storoni L.C., Read R.J. (2007). Phaser crystallographic software. J. Appl. Crystallogr..

[32] Emsley P., Lohkamp B., Scott W.G., Cowtan K. (2010). Features and development of Coot. Acta Crystallogr. D.

[33] Afonine P.V., Grosse-Kunstleve R.W., Echols N., Headd J.J., Moriarty N.W., Mustyakimov M., Terwilliger T.C., Urzhumtsev A. (2012). Towards automated crystallographic structure refinement with phenix.refine. Acta Crystallogr. D.

[34] Chen V.B., Arendall W.B., Headd J.J., Keedy D.A., Immormino R.M., Kapral G.J., Murray L.W., Richardson J.S. (2010). MolProbity: all-atom structure validation for macromolecular crystallography. Acta Crystallogr. D.

[35] Sievers F., Wilm A., Dineen D., Gibson T.J., Karplus K., Li W., Lopez R., McWilliam H. (2011). Fast, scalable generation of high-quality protein multiple sequence alignments using Clustal Omega. Mol. Syst. Biol..

